# Metabolic syndrome and cardiovascular disease in cancer survivors

**DOI:** 10.1002/jcsm.13443

**Published:** 2024-03-22

**Authors:** Kensuke Ueno, Hidehiro Kaneko, Yuta Suzuki, Akira Okada, Satoshi Matsuoka, Katsuhito Fujiu, Nobuaki Michihata, Taisuke Jo, Norifumi Takeda, Hiroyuki Morita, Kentaro Kamiya, Junya Ako, Koichi Node, Hideo Yasunaga, Issei Komuro

**Affiliations:** ^1^ Department of Cardiovascular Medicine University of Tokyo Tokyo Japan; ^2^ Department of Rehabilitation Sciences, Graduate School of Medical Sciences Kitasato University Kanagawa Japan; ^3^ Department of Advanced Cardiology University of Tokyo Tokyo Japan; ^4^ Department of Prevention of Diabetes and Lifestyle‐Related Diseases, Graduate School of Medicine University of Tokyo Tokyo Japan; ^5^ Department of Health Services Research University of Tokyo Tokyo Japan; ^6^ Department of Rehabilitation, School of Allied Health Sciences Kitasato University Kanagawa Japan; ^7^ Department of Cardiovascular Medicine, School of Medicine Kitasato University Kanagawa Japan; ^8^ Department of Cardiovascular Medicine Saga University Saga Japan; ^9^ Department of Clinical Epidemiology and Health Economics, School of Public Health University of Tokyo Tokyo Japan

**Keywords:** Cancer survivors, Cardiovascular disease, Epidemiology, Metabolic syndrome, Onco‐cardiology

## Abstract

**Background:**

The risk of subsequent cardiovascular disease (CVD) is high in cancer survivors. Although metabolic syndrome is an established risk factor for CVD, its association with cancer survivors has not yet been established. This study aimed to clarify whether metabolic syndrome is associated with subsequent CVD risk in patients with cancer using a nationwide epidemiological dataset.

**Methods:**

We retrospectively analysed 53 510 patients with a history of breast, colorectal, or stomach cancer, which is reportedly a major site for developing cancer in Japan. Study participants were categorized into two groups based on the presence of metabolic syndrome, defined using the Japanese criteria (high waist circumference and ≥2 metabolic parameters including elevated blood pressure, elevated triglycerides, reduced high‐density lipoprotein cholesterol, or elevated fasting plasma glucose). The clinical outcomes were collected between 2005 and 2021. The primary endpoint was defined as the composite CVD outcome, including myocardial infarction, angina pectoris, stroke, and heart failure.

**Results:**

The median patient age was 54 years, and 37.5% of the patients were men. Metabolic syndrome was observed in 5558 (10.4%) patients. Over a mean follow‐up period of 973 ± 791 days, 3085 composite CVD outcomes were recorded. Multivariable Cox regression analyses showed that metabolic syndrome was associated with a greater risk of developing CVD events (HR = 1.29, 95% CI = 1.15–1.45). Metabolic syndrome was also associated with an increased risk of CVD in patients with a follow‐up period ≥1 year (HR = 1.33, 95% CI = 1.15–1.53). This relationship was also observed when metabolic syndrome was defined according to the International Diabetes Federation criteria (HR = 1.34, 95% CI = 1.21–1.49) and the National Cholesterol Education Program Adult Treatment Panel III criteria (HR = 1.32, 95% CI = 1.19–1.46). Subgroup analyses showed that the relationship between metabolic syndrome and incident CVD was more pronounced in the non‐obese participants than in the obese participants.

**Conclusions:**

Metabolic syndrome is associated with a greater risk of developing CVD, even among cancer survivors.

## Introduction

Metabolic syndrome increases the risk of developing cardiovascular disease (CVD).^1^, ^2^, ^3^ For example, a meta‐analysis including 43 cohorts and 172 573 individuals showed that the presence of metabolic syndrome had a relative risk of CV events and death of 1.78 (95% confidence interval [CI] = 1.58–2.00).^3^ Further, metabolic syndrome may increase the risk of developing several cancers.^4^, ^5^ We previously reported that the presence of metabolic syndrome was linked to an increased risk of colorectal cancer even in young adults (hazard ratio 1.26, 95% CI = 1.07–1.49).^5^ Accordingly, the importance of metabolic syndrome is attracting clinical interest in the field of both cardiovascular medicine and oncology. Recent studies have indicated that cancer survivors have a higher CVD risk than individuals without cancer, which is an emerging clinical issue in onco‐cardiology. However, it remains unclear whether metabolic syndrome influences the subsequent risk of CVD events in cancer survivors. In this study, we examined the association between the presence of metabolic syndrome and subsequent risk of developing CVD in patients with cancer. We also investigated whether the definition of metabolic syndrome affects the connection between metabolic syndrome and incident CVD among cancer survivors.

## Methods

Anyone who purchases the JMDC claims database from JMDC Inc. (https://www.jmdc.co.jp/en) can use this database.

### Study population

We retrospectively analysed the JMDC Claims Database (Tokyo, Japan), which includes health check‐up and insurance claims records obtained from >60 insurers between 2005 and 2021.^6^, ^7^, ^8^ Administrative claims data (e.g., cancer diagnosis and CVD diagnosis) were recorded using the International Classification of Diseases, 10^th^ Revision (ICD‐10) codes. From this dataset, we extracted 67 480 patients with diagnoses of breast (ICD‐10: C50), colorectal (ICD‐10: C18–20), or stomach (ICD‐10: C16) cancers who underwent a health check‐up with information for the diagnosis of metabolic syndrome after the diagnosis of cancer >1 year after insurance enrollment (1‐year look‐back period) (Figure S1). We excluded 5482 individuals with a history of CVD, 48 individuals with a history of renal replacement therapy, and individuals with missing data on body mass index (BMI) (*n* = 11), low‐density lipoprotein cholesterol (*n* = 15), alcohol consumption (*n* = 6535), cigarette smoking (*n* = 7), or physical activity (n = 1872). Finally, 53 510 participants were included in this study (Figure S2).

### Ethics

According to the principles of the Declaration of Helsinki, the present study was performed based on the ethical guidelines of the University of Tokyo (approved by the Ethical Committee of the University of Tokyo: 2018‐10862). Because all data in this dataset were de‐identified, the requirement for informed consent was waived. All data complied with the International Conference on Harmonization guidelines.^9^

### Metabolic syndrome and other measurements

Health check‐up data were obtained using standardized protocols. Regarding the diagnostic criteria for metabolic syndrome in Japan,^10^, ^11^ high waist circumference, defined as waist circumference at the level of umbilical ≥85 cm in men and ≥90 cm in women, is mandatory, and any two or three of the following abnormalities are required: high blood pressure (systolic blood pressure ≥130 mmHg or diastolic blood pressure ≥85 mmHg) or use of antihypertensive medications, hyperglycaemia (fasting plasma glucose level ≥110 mg/dL or use of antidiabetic medications), or dyslipidaemia (triglyceride level ≥150 mg/dL or high‐density lipoprotein cholesterol level <40 mg/dL, or use of lipid‐lowering medications). The International Diabetes Federation (IDF)^12^ and National Cholesterol Education Program Adult Treatment Panel III (NCEP/ATP III) criteria are summarized in Table S1.^13^ We obtained information on cigarette smoking (current or non‐current) and alcohol consumption (every day or not every day) from the self‐reported questionnaires as previously described.^5^ We defined obesity as BMI ≥ 25 kg/m^2^. Physical inactivity was defined as applying to individuals who did not report engaging in 30 min of exercise ≥twice a week or walking ≥1 h per day based on a questionnaire used at a health check‐up as previously described.^6^

### Outcomes

Data on the outcomes that occurred between January 2005 and April 2021 were collected. A composite endpoint that included myocardial infarction (MI) defined as ICD‐10 codes I210–I214 and I219; angina pectoris (AP) defined as ICD‐10 codes I200, I201, I208, and I209; stroke defined as ICD‐10 codes I630, I631–I636, I638, I639, I600–I611, I613–I616, I619, I629, and G459; and heart failure (HF), defined as ICD‐10 codes I500, I501, I509, and I110, was defined as the primary outcome. As a secondary outcome, the relationship between metabolic syndrome and the risk for each of MI, AP, stroke, and HF events was examined individually.

### Statistical analysis

Summary statistics for the clinical characteristics of the patients with and without metabolic syndrome were calculated. The Wilcoxon rank‐sum test was used to compare the continuous variables. Chi‐square tests were performed to compare categorical variables. The cumulative incidence of CVD events was compared between patients with and without metabolic syndrome using Kaplan–Meier curves and log‐rank tests. Cox regression analyses were conducted to identify the relationship between the presence of metabolic syndrome and the subsequent risk of incident CVD. Model 1 included only metabolic syndrome (unadjusted model); model 2 included metabolic syndrome, age, and sex; and model 3 included metabolic syndrome, age, sex, BMI, low‐density lipoprotein cholesterol level, current cigarette smoking, alcohol drinking, physical inactivity, cancer sites, and active cancer treatment before and after 6 months.

As the threshold of waist circumference for the diagnosis of metabolic syndrome differed between sexes, the link between metabolic syndrome and incident CVD was examined separately by sex. *P*‐values were calculated for interactions between the sexes. The relationship between each component of the diagnostic criteria for metabolic syndrome and the subsequent risk of CVD was analysed using the Cox regression model.

Five sensitivity analyses were performed.

First, the relationship between metabolic syndrome and incident CVD was examined using the IDF criteria for Asians and NCEP/ATP III criteria and also separately analysed in men and women.

Second, based on the assumption that missing data occurred at random, missing values for covariates were imputed using multiple imputations with chained equations and 20 iterations. Rubin's rules were used to derive hazard ratios (HRs) and standard errors.

Third, patients whose follow‐up period for CVD was shorter than 1 year were eliminated to reduce the potential impact of latent CVD.

Fourth, death could be a competing risk for CVD events; thus, we performed a competing risks analysis. The deaths collected in this study were all‐cause deaths.

Fifth, to perform subgroup analysis, we divided the study participants by age (≥50 years and <50 years), obesity, active cancer treatment before and after 6 months and 12 months, and cancer sites, and conducted multivariable Cox regression analyses. *P*‐values for the interactions were calculated using a multivariable model. Statistical significance was defined as a *P*‐value < 0.05. Regarding the interaction analyses, a *P*‐value for interaction < 0.10 was considered statistically significant. All statistical analyses were conducted using the Stata software (version 17; StataCorp LLC, College Station, TX, USA).

## Results

### Background characteristics

The clinical characteristics of the participants are presented in Table 1. Among the study population, 25 167 (47.0%), 18 379 (34.3%), and 11 115 (20.8%) patients had a history of breast cancer, colorectal cancer, and stomach cancer, respectively; 1131 (2.1%) had a history of two cancers, and 10 (<0.1%) had a history of three cancers. The median age of the patients was 54 (IQR, 48–61) years; 20 093 patients (37.5%) were men, and 5558 patients (10.4%) had metabolic syndrome. Patients meeting the criteria for metabolic syndrome were older and more likely to be men, obese, cigarette smokers (current), alcohol drinkers (every day), and physically inactive than those without metabolic syndrome.

**Table 1:** Characteristics of study participants

	Metabolic syndrome (−)	Metabolic syndrome (+)	*P*‐value
	*n* = 47 952	*n* = 5558	
Stomach cancer, *n* (%)	9814 (20.5)	1301 (23.4)	<0.001
Colorectal cancer, *n* (%)	15 101 (31.5)	3278 (59.0)	<0.001
Breast cancer, *n* (%)	24 062 (50.2)	1105 (19.9)	<0.001
Number of cancers			
1	46 937 (97.9)	5432 (97.7)	0.390
2	1005 (2.1)	126 (2.3)	
3	10 (0.0)	0 (0.0)	
Age, years	54 (48–60)	58 (53–63)	<0.001
Men, *n* (%)	15 978 (33.3)	4115 (74.0)	<0.001
Body mass index, kg/m^2^	21.5 (19.6–23.6)	26.7 (24.8–29.1)	<0.001
Waist circumference, cm	78.5 (72.5–84.0)	93.2 (89.5–98.3)	<0.001
Systolic blood pressure, mmHg	118 (107–129)	134 (125–143)	<0.001
Diastolic blood pressure, mmHg	73 (66–81)	83 (76–90)	<0.001
Fasting plasma glucose, mg/dL	93 (87–100)	111 (98–124)	<0.001
Low‐density lipoprotein cholesterol, mg/dL	118 (97–140)	122 (101–144)	<0.001
High‐density lipoprotein cholesterol, mg/dL	69 (58–81)	53 (45–62)	<0.001
Triglycerides, mg/dL	78 (57–110)	157 (106–210)	<0.001
Alcohol drinking, *n* (%)	9864 (20.6)	1828 (32.9)	<0.001
Current cigarette smoking, *n* (%)	5710 (11.9)	1176 (21.2)	<0.001
Physical inactivity, *n* (%)	24 848 (51.8)	3187 (57.3)	<0.001

For the diagnostic criteria for metabolic syndrome in Japan, high waist circumference, defined as waist circumference at umbilical level ≥85 cm in men and ≥90 cm in women, was obligatory, and any two or three of the following abnormalities were required: high blood pressure (systolic blood pressure ≥130 mmHg or diastolic blood pressure ≥85 mmHg) or use of antihypertensive medications, hyperglycaemia (fasting plasma glucose level ≥110 mg/dL or use of antidiabetic medications), or dyslipidaemia (triglycerides level ≥150 mg/dL or high‐density lipoprotein cholesterol level <40 mg/dL, or use of lipid‐lowering medications).

### Metabolic syndrome and composite cardiovascular disease endpoint

**Figure 1:**
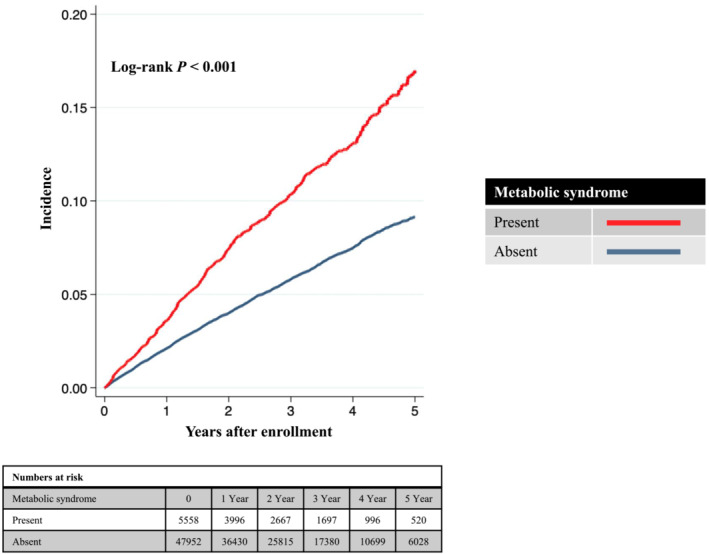
Kaplan–Meier curves. The study participants were categorized into two groups based on the presence of metabolic syndrome. The cumulative probability of cardiovascular events was calculated using the Kaplan–Meier method. The log‐rank test was used to calculate the *P*‐value, which was <0.001.

During a mean follow‐up period of 973 ± 791 days, 3085 CVD events occurred. Kaplan–Meier curves demonstrated a significant difference in cumulative CVD incidence between patients with and without metabolic syndrome (Figure 1). The incidence rates for CVD were higher in patients with metabolic syndrome (368.2 [95% CI = 336.9–402.3] per 10 000 person‐years) than in those without metabolic syndrome (200.8 [95% CI = 193.2–208.7] per 10 000 person‐years). Univariate (Model 1) and age‐sex‐adjusted (Model 2) Cox regression analyses showed that the prevalence of metabolic syndrome was associated with a higher risk of developing CVD. Multivariable Cox regression analysis (Model 3) demonstrated that the presence of metabolic syndrome was associated with a greater CVD risk (HR = 1.29, 95% CI = 1.15–1.45) (Table 2).

**Table 2:** The frequency of events, corresponding incidence rates, and hazard ratios for cardiovascular disease events among participants by metabolic syndrome

Variable	Overall		Men		Women	
	Metabolic syndrome		Metabolic syndrome		Metabolic syndrome	
	Absent	Present	Absent	Present	Absent	Present
Number	47 952	5558	15 978	4115	31 974	1443
CVD events	2596	489	1074	384	1522	105
Incidence rate (95% CI)	200.8 (193.2–208.7)	368.2 (336.9–402.3)	240.6 (226.6–255.4)	382.8 (346.4–423.1)	179.8 (171.0–189.1)	323.0 (266.8–391.1)
Model 1 (Unadjusted)	1 [Reference]	1.83 (1.66–2.01)	1 [Reference]	1.59 (1.41–1.78)	1 [Reference]	1.79 (1.47–2.18)
Model 2	1 [Reference]	1.47 (1.32–1.62)	1 [Reference]	1.48 (1.32–1.66)	1 [Reference]	1.43 (1.17–1.74)
Model 3	1 [Reference]	1.29 (1.15–1.45)	1 [Reference]	1.43 (1.24–1.64)	1 [Reference]	1.09 (0.87–1.36)

The incidence rate was per 10 000 person‐years. Unadjusted and adjusted hazard ratios (95% CI) associated with metabolic syndrome are shown. Model 1 is unadjusted. Model 2 includes adjustment for age and sex. Model 3 includes adjustment for age, sex, body mass index, low‐density lipoprotein cholesterol level, current cigarette smoking, alcohol drinking, physical inactivity, cancer sites, and active cancer treatment before and after 6 months. In the analysis stratified by sex, sex was excluded from covariates. Patients were categorized into two groups according to the absence or presence of metabolic syndrome.

CI = confidence interval; CVD = cardiovascular disease.

### Metabolic syndrome and each cardiovascular event

During the follow‐up, 1547 HF, 120 MI, 1307 AP, and 702 stroke events were recorded. In multivariable adjustment models (Model 3), co‐morbid metabolic syndrome was associated with a higher risk of HF (HR = 1.24, 95% CI = 1.05–1.45), MI (HR = 2.01, 95% CI = 1.20–3.39), AP (HR = 1.36, 95% CI = 1.14–1.62), and stroke (HR = 1.43, 95% CI = 1.13–1.82) (Table S2).

### Components of metabolic syndrome and cardiovascular event

High blood pressure, high blood glucose level, and high waist circumference were associated with incident CVD, but dyslipidaemia was not. In men, high blood pressure, high blood glucose, and high waist circumference were associated with incident CVD, whereas high blood pressure and high blood glucose were associated with incident CVD in women (Table 3). The *P*‐values for the interaction between sexes were 0.83, 0.53, 0.44, and 0.73 for high waist circumference, high blood pressure, dyslipidaemia, and high blood glucose, respectively, indicating that the association between each component of metabolic syndrome and incident CVD would not be modified by sex.

**Table 3:** Association of each component of the criteria for metabolic syndrome with incident cardiovascular disease

	Overall	Men	Women
High waist circumference^a^	1.15 (1.03–1.29)	1.26 (1.09–1.46)	1.05 (0.87–1.26)
High blood pressure^b^	1.44 (1.33–1.56)	1.50 (1.33–1.68)	1.40 (1.25–1.56)
Dyslipidaemia^c^	1.05 (0.96–1.14)	1.03 (0.92–1.15)	1.07 (0.94–1.21)
High blood glucose^d^	1.19 (1.08–1.31)	1.18 (1.04–1.33)	1.21 (1.02–1.42)

Hazard ratios (95% confidence interval) of high waist circumference, high blood pressure, dyslipidaemia, and high blood glucose were adjusted for age, sex, body mass index, low‐density lipoprotein cholesterol level, cigarette smoking, alcohol consumption, physical inactivity, cancer sites, and active cancer treatment before and after 6 months in the population model. Hazard ratios of high waist circumference, high blood pressure, dyslipidaemia, and high blood glucose were adjusted for age, body mass index, low‐density lipoprotein cholesterol level, cigarette smoking, alcohol consumption, physical inactivity, cancer sites, and active cancer treatment before and after 6 months in men and women.

^a^ Waist circumference at umbilical level ≥85 cm in men and ≥90 cm in women.

^b^ Systolic blood pressure ≥130 mmHg, diastolic blood pressure ≥85 mmHg or use of antihypertensive medications.

^c^ Triglycerides level ≥150 mg/dL or high‐density lipoprotein cholesterol level <40 mg/dL, or use of lipid‐lowering medications.

^d^ Fasting plasma glucose level ≥110 mg/dL or use of antidiabetic medications.

### Stratified analysis by sex

Study participants were stratified by sex. Unadjusted Cox regression analyses showed that the prevalence of metabolic syndrome was associated with a higher risk of CVD in both men and women. Multivariable Cox regression analyses showed that metabolic syndrome was associated with an elevated risk of developing CVD in men (HR = 1.43, 95% CI = 1.24–1.64) but not in women (HR = 1.09, 95% CI = 0.87–1.36, *P* for interaction = 0.33) (Table 2).

### Sensitivity analyses

The association between metabolic syndrome and incident CVD was evaluated using different diagnostic criteria for metabolic syndrome. Using the IDF criteria, metabolic syndrome was linked to an increased risk of incident CVD (HR = 1.34, 95% CI = 1.21–1.49) (Table S3). This was related to the incidence of CVD in both sexes (*P* for interaction = 0.64) (Table S3). Consistent results were obtained using the NCEP/ATP III criteria (HR = 1.32, 95% CI = 1.19–1.46). The presence of metabolic syndrome was associated with subsequent risk of CVD in both men and women (*P* for interaction = 0.49) (Table S4).

**Figure 2:**
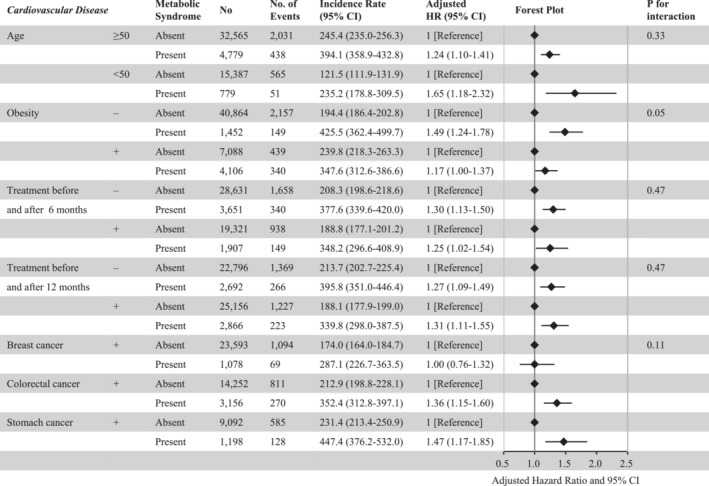
Subgroup analyses. The incidence rate was per 10 000 person‐years. For each subgroup, we conducted a cox regression analysis. Adjusted for age, sex, body mass index, low‐density lipoprotein cholesterol level, current cigarette smoking, alcohol drinking, physical inactivity, cancer sites, and active cancer treatment before and after 6 months in the subgroup analyses stratified by age ≥50 or <50 years. Adjusted for age, sex, body mass index, low‐density lipoprotein cholesterol level, current cigarette smoking, alcohol consumption, physical inactivity, cancer sites, and active cancer treatment before and after 6 months in the subgroup analyses stratified by obesity. Adjusted for age, sex, body mass index, low‐density lipoprotein cholesterol level, current cigarette smoking, alcohol drinking, physical inactivity, and cancer sites in the subgroup analyses stratified by active cancer treatment before and after 6 months. Adjusted for age, sex, body mass index, low‐density lipoprotein cholesterol level, current cigarette smoking, alcohol consumption, physical inactivity, and cancer sites in the subgroup analyses stratified by active cancer treatment before and after 12 months. Adjusted for age, sex, body mass index, low‐density lipoprotein cholesterol level, current cigarette smoking, alcohol consumption, physical inactivity, and active cancer treatment before and after 6 months in the subgroup analyses stratified by cancer site. Hazard ratios (95% confidence intervals) are also presented. CI, confidence interval; HR, hazard ratio.

Participants with metabolic syndrome had a greater risk of developing CVD after multiple imputations for missing data (HR = 1.31, 95% CI = 1.18–1.46) (Table S5). We excluded 13 060 participants with a follow‐up period shorter than 1 year and analysed 40 450 participants. In this population, metabolic syndrome was associated with a higher risk of developing CVD (HR = 1.33, 95% CI = 1.15–1.53) (Table S6). The relationship between metabolic syndrome and incident CVD did not change in the competing risks model (Table S7). The association of metabolic syndrome with subsequent risk for CVD events was consistent across the subgroups. Interaction analysis indicated that this association was more pronounced in non‐obese participants than in obese participants (Figure 2). In analyses stratified by respective cancers, metabolic syndrome was associated with a higher risk of developing CVD in patients with colorectal cancer (HR = 1.36, 95% CI = 1.15–1.60) and stomach cancer (HR = 1.47, 95% CI = 1.17–1.85), but not in breast cancer patients (HR = 1.00, 95% CI = 0.76–1.32) (Figure 2).

## Discussion

Using a large‐scale health check‐up and insurance claims dataset, we studied more than 50 000 patients with a history of breast, colorectal, or stomach cancer. Patients with cancer diagnosed with metabolic syndrome had a greater risk of developing CVD; this relationship was unchanged irrespective of the diagnostic criteria for metabolic syndrome.

Cancer survivors have been reported to have a higher risk of developing CVD compared with individuals without cancer,^14^, ^15^, ^16^ and there is a need to identify factors associated with the development of CVD in cancer survivors. The development of metabolic syndrome is associated with signs of early atherosclerosis and may contribute to an increased risk of CVD, in addition to direct CV toxicity from some cancer treatments.^17^ In addition, prolonged survival of cancer survivors would be expected to increase the prevalence of metabolic syndrome and prolong the duration of the disease, which may lead to further attraction of CVD development. Notably, previous studies reported that while 34.1% of cancer survivors actually had metabolic syndrome, only a mere 6.8% were diagnosed with it.^18^ Considering these backgrounds, metabolic syndrome may be an associated factor in the development of CVD even in cancer survivors, and it is important to identify metabolic syndrome early in cancer survivors and lead to appropriate treatment. Nevertheless, the association between metabolic syndrome and CVD development in cancer survivors has not yet been established. This is the first study to show that metabolic syndrome is associated with an increased risk of developing CVD, even in cancer survivors.

Metabolic syndrome, which is a cluster of metabolic abnormalities predisposing to central adiposity, is an established risk factor for developing CVD. Although the prevalence was different depending on the cancer site,^17^ metabolic syndrome was not a rare co‐morbidity among cancer survivors. Further, preceding studies indicated that co‐morbid metabolic syndrome potentially worsened clinical outcomes of cancer patients.^19^, ^20^ However, to date, data comparing the subsequent CVD risk between cancer patients with and without metabolic syndrome are scarce.

The primary findings of this study are globally consistent with those of previous studies. In our study, metabolic syndrome was observed in 10.4% of participants. Metabolic syndrome was prevalent in 20.5% of male participants. When we used the IDF or NCEP/ATP III criteria, the percentages of metabolic syndrome were 14.5% and 13.6%, respectively. As expected, the participants with metabolic syndrome had worse background characteristics. However, even after adjusting for covariates, the presence of metabolic syndrome was linked to a greater risk of CVD. Several sensitivity analyses confirmed the robustness of our results. The relationship between metabolic syndrome and incident CVD existed even when using the IDF or NCEP/ATP IIII criteria.

In the present study, the results of subgroup analyses stratified by sex and cancer type were not consistent. In men, metabolic syndrome was associated with a higher risk of developing CVD, but not in women. In with colorectal and stomach cancer, metabolic syndrome was associated with a higher risk of developing CVD, but not in those having breast cancer. Given that most patients having breast cancer were women, the difference in our results stratified by cancer site would be attributable to sex difference. On the other hand, when metabolic syndrome was defined using the International Diabetes Federation and National Cholesterol Education Program Adult Treatment Panel III criteria, metabolic syndrome was associated with a higher risk of developing CVD even in women. Thus, the association between metabolic syndrome and incident CVD in women would change depending on the definition of metabolic syndrome. Further studies are needed to validate the results of our study.

Notably, subgroup analyses showed that the association between metabolic syndrome and future risk of CVD development was more pronounced in non‐obese participants than in obese participants. The coexistence of abdominal obesity and metabolic abnormalities despite normal weight may increase the risk of CVD in patients with cancer. We previously reported the pathological relationship between normal‐weight central obesity and the subsequent risk of developing CVD in the general population.^21^ Our results showed that normal‐weight central obesity is also important in cancer survivors. Our findings would also imply a potential role for sarcopenic obesity, which has recently attracted clinical interest in CVD and cancer.^22^ Further studies using definitions based on BMI and waist circumference are needed to clarify the detailed association between normal‐weight central obesity and the development of CVD in cancer survivors. However, this finding of our study suggests the importance of assessing metabolic syndrome, even if cancer survivors are not obese. There are possible reasons underlying the increased risk of CVD in the presence of metabolic syndrome even at a normal weight. First, BMI and WC may provide different information. Although BMI and WC are relatively highly correlated,^23^ the combination of BMI and WC has been reported to be more useful in predicting fat distribution than BMI or WC alone.^24^ It is also well established that WC is an important predictor of metabolic abnormalities and CVD development even after controlling for BMI.^21^, ^25^, ^26^ Furthermore, the presence of abdominal obesity, even at normal weight, has been reported to increase the risk of developing CVD and CVD mortality.^21^, ^23^, ^26^ BMI is difficult to distinguish between fat mass and skeletal muscle mass. Therefore, high WC, even at normal weight, may reflect excess visceral fat and low muscle mass (i.e., sarcopenic obesity), which may increase CVD risk. Among cancer patients with normal weight but central obesity, these facts remind us to consider a possibility of skeletal muscle loss concomitant with visceral fat accumulation, that is, sarcopenic obesity. Second, it has been reported that excess visceral fat is associated with hypertension, dyslipidaemia, and diabetes mellitus.^27^, ^28^ As central obesity and metabolic abnormalities are traditional risk factors for developing CVD, their coexistence can be considered to increase the risk of developing CVD additively. Third, based on BMI, overweight and obese individuals may have more subcutaneous fat in their hips and legs, which is associated with a healthier metabolic profile.^29^ These factors may have increased the risk of developing CVD in patients with cancer in the presence of central obesity despite the normal weight, but further studies are needed to clarify the detailed mechanisms.

Our study has several clinical implications. Given that the clinical importance of CVD in cancer survivors is steadily increasing, CVD risk stratification among individuals with cancer must be established. From this perspective, our results indicate that the assessment of metabolic syndrome could identify cancer survivors at a high risk for subsequent CVD events. Further studies are required to uncover whether intervention (or treatment) for metabolic syndrome would prevent CVD development in cancer patients. According to the hazard ratio(s) of each component of metabolic syndrome, normalizing blood pressure would be the most promising. Moreover, various factors, such as lifestyle, cancer treatment, and hormonal disorders, could contribute to the pathology of metabolic syndrome in cancer patients; therefore, a detailed assessment of the aetiology of metabolic syndrome is needed. Lastly, it is important for physicians to recognize that the presence of metabolic syndrome could increase CVD risk even in cancer patients, and the clinical importance of this condition should not be underestimated from the perspective of CVD prevention.

In this study, a nationwide longitudinal health check‐up dataset with high outcome ascertainment rates was used, which is strong because of its electronic connection to administrative claims records. However, the present study had some limitations, and most limitations are due to using this health check‐up and claims database as previously described.^21^, ^30^ Even after multivariable analyses, the possibility of residual confounding factors, such as diet, insulin resistance, and socioeconomic status could not be eliminated. Patients with a history of the three cancer types were included in this study because the prevalence of these three cancers is common in Japan (https://ganjoho.jp/reg_stat/statistics/stat/summary.html). Nevertheless, whether our primary findings, indicating a potential link between the presence of metabolic syndrome and a greater risk of developing CVD, could be applicable to patients with other cancer types remains unknown. The accuracy (particularly specificity) of disease diagnoses recorded in the Japanese claims database was reported to be high,^31^, ^32^ and the CVD incidence in our dataset was comparable to that of other epidemiological data in Japan.^33^, ^34^ Thus, we believe that our analysis could reflect real‐world settings in Japan. However, recorded diagnoses in the administrative claims database should generally be considered less validated. Given that the JMDC Claims Database does not include individuals aged >75 years, it is unknown whether our findings can be expanded to elderly cancer patients. Our dataset lacks detailed information on cancer (i.e., cancer stage). Although the association between metabolic syndrome and a greater CVD risk does not change in a competing risks model, cancer status could undoubtedly influence our study results. While it is widely known that the metabolic syndrome is closely associated with an elevated risk for developing CVD in general population, this study shows that the metabolic syndrome could also be related to an increased risk for CVD development in cancer patients. Future investigations are needed to examine whether the association could be altered by the presence of cancer.

In conclusion, the present analysis of a large‐scale epidemiological database including over 50 000 cancer survivors showed that metabolic syndrome was associated with a greater risk of developing CVD, suggesting that the assessment of metabolic abnormalities would be clinically helpful for CVD risk stratification among patients living with cancer.

## Funding

This work was supported by grants from the Ministry of Health, Labour and Welfare, Japan (21AA2007) and the Ministry of Education, Culture, Sports, Science and Technology, Japan (20H03907, 21H03159, and 21K08123). The funding sources had nothing regarding the current study.

## Conflict of interest

Research funding and scholarship funds were provided (Hidehiro Kaneko and Katsuhito Fujiu) by Medtronic Japan Co., Ltd, Abbott Medical Japan Co., Ltd, Boston Scientific Japan Co., Ltd, and Fukuda Denshi, Central Tokyo Co., Ltd. Other authors received no funding and have no conflicts of interest to declare.

## Supporting information


**Table S1.** The International Diabetes Federation's criteria and the National Cholesterol Education Program Adult Treatment Panel III criteria for defining metabolic syndrome.
**Table S2.** Metabolic Syndrome and Each Cardiovascular Event.
**Table S3.** The Frequency of Events, Corresponding Incidence Rates, and Hazard Ratios for Cardiovascular Disease Events Among Participants by Metabolic Syndrome Defined using the International Diabetes Federation criteria.
**Table S4.** The Frequency of Events, Corresponding Incidence Rates, and Hazard Ratios for Cardiovascular Disease Events Among Participants by Metabolic Syndrome Defined using the National Cholesterol Education Program Adult Treatment Panel III criteria.
**Table S5.** Multiple Imputation.
**Table S6.** Analysis after the exclusion of subjects with follow‐up period shorter than one year.
**Table S7.** Competing Risks Analysis.

**Figure S1.** Study Design.
**Figure S2.** Flowchart.

## References

[1] Ford ES . Risks for all‐cause mortality, cardiovascular disease, and diabetes associated with the metabolic syndrome: a summary of the evidence. Diabetes Care 2005;28:1769–1778. 15983333 10.2337/diacare.28.7.1769

[2] Galassi A , Reynolds K , He J . Metabolic syndrome and risk of cardiovascular disease: a meta‐analysis. Am J Med 2006;119:812–819. 17000207 10.1016/j.amjmed.2006.02.031

[3] Gami AS , Witt BJ , Howard DE , Erwin PJ , Gami LA , Somers VK , et al. Metabolic syndrome and risk of incident cardiovascular events and death: a systematic review and meta‐analysis of longitudinal studies. J Am Coll Cardiol 2007;49:403–414. 17258085 10.1016/j.jacc.2006.09.032

[4] Esposito K , Chiodini P , Colao A , Lenzi A , Giugliano D . Metabolic syndrome and risk of cancer: a systematic review and meta‐analysis. Diabetes Care 2012;35:2402–2411. 23093685 10.2337/dc12-0336 PMC3476894

[5] Jimba T , Kaneko H , Yano Y , Itoh H , Yotsumoto H , Seki H , et al. Relation of the metabolic syndrome to incident colorectal cancer in young adults aged 20 to 49 years. Am J Cardiol 2021;158:132–138. 34481589 10.1016/j.amjcard.2021.07.049

[6] Kaneko H , Itoh H , Kamon T , Fujiu K , Morita K , Michihata N , et al. Association of cardiovascular health metrics with subsequent cardiovascular disease in young adults. J Am Coll Cardiol 2020;76:2414–2416. 33183514 10.1016/j.jacc.2020.09.545

[7] Kaneko H , Yano Y , Itoh H , Morita K , Kiriyama H , Kamon T , et al. Association of blood pressure classification using the 2017 American College of Cardiology/American Heart Association blood pressure guideline with risk of heart failure and atrial fibrillation. Circulation 2021;143:2244–2253. 33886370 10.1161/CIRCULATIONAHA.120.052624

[8] Suzuki Y , Kaneko H , Okada A , Matsuoka S , Fujiu K , Michihata N , et al. Kidney outcomes in patients with diabetes mellitus did not differ between individual sodium‐glucose cotransporter‐2 inhibitors. Kidney Int 2022;102:1147–1153. 35961884 10.1016/j.kint.2022.05.031

[9] Dixon JR Jr . The international conference on harmonization good clinical practice guideline. Qual Assur 1998;6:65–74. 10386329 10.1080/105294199277860

[10] Itoh H , Kaneko H , Kiriyama H , Yoshida Y , Nakanishi K , Mizuno Y , et al. Effect of metabolically healthy obesity on the development of carotid plaque in the general population: a community‐based cohort study. J Atheroscler Thromb 2020;27:155–163. 31231080 10.5551/jat.48728 PMC7049475

[11] Matsuzawa Y . Metabolic syndrome ‐ definition and diagnostic criteria in Japan. J Atheroscler Thromb 2005;12:301. 16394611 10.5551/jat.12.301

[12] Alberti KG , Zimmet P , Shaw J . Metabolic syndrome—a new world‐wide definition. A consensus statement from the International Diabetes Federation. Diabet Med 2006;23:469–480. 16681555 10.1111/j.1464-5491.2006.01858.x

[13] Grundy SM , Cleeman JI , Daniels SR , Donato KA , Eckel RH , Franklin BA , et al. Diagnosis and management of the metabolic syndrome: an American Heart Association/National Heart, Lung, and Blood Institute Scientific Statement. Circulation 2005;112:2735–2752. 16157765 10.1161/CIRCULATIONAHA.105.169404

[14] Sturgeon KM , Deng L , Bluethmann SM , Zhou S , Trifiletti DM , Jiang C , et al. A population‐based study of cardiovascular disease mortality risk in US cancer patients. Eur Heart J 2019;40:3889–3897. 31761945 10.1093/eurheartj/ehz766 PMC6925383

[15] Schoormans D , Vissers PAJ , van Herk‐Sukel MPP , Denollet J , Pedersen SS , Dalton SO , et al. Incidence of cardiovascular disease up to 13 year after cancer diagnosis: a matched cohort study among 32 757 cancer survivors. Cancer Med 2018;7:4952–4963. 30220107 10.1002/cam4.1754 PMC6198235

[16] Florido R , Daya NR , Ndumele CE , Koton S , Russell SD , Prizment A , et al. Cardiovascular disease risk among cancer survivors: the Atherosclerosis Risk In Communities (ARIC) Study. J Am Coll Cardiol 2022;80:22–32. 35772913 10.1016/j.jacc.2022.04.042 PMC9638987

[17] de Haas EC , Oosting SF , Lefrandt JD , Wolffenbuttel BH , Sleijfer DT , Gietema JA . The metabolic syndrome in cancer survivors. Lancet Oncol 2010;11:193–203. 20152771 10.1016/S1470-2045(09)70287-6

[18] Seo Y , Kim JS , Park ES , Ryu E . Assessment of the awareness and knowledge of cancer survivors regarding the components of metabolic syndrome. PLoS ONE 2018;13:e0199142. 29920529 10.1371/journal.pone.0199142 PMC6007835

[19] Yang X , Li X , Dong Y , Fan Y , Cheng Y , Zhai L , et al. Effects of metabolic syndrome and its components on the prognosis of endometrial cancer. Front Endocrinol (Lausanne) 2021;12:780769. 34975754 10.3389/fendo.2021.780769 PMC8717682

[20] Awwad F , Ozga AK , Amin T , Schlueter C , Kailani S , Perez D , et al. Metabolic syndrome is associated with impaired survival after surgery for pancreatic neuroendocrine tumors. Neuroendocrinology 2022;112:1225–1236. 35354139 10.1159/000524366

[21] Ueno K , Kaneko H , Kamiya K , Itoh H , Okada A , Suzuki Y , et al. Relationship of normal‐weight central obesity with the risk for heart failure and atrial fibrillation: analysis of a nationwide health check‐up and claims database. Eur Heart J Open 2022;2:oeac026. 35919350 10.1093/ehjopen/oeac026 PMC9242061

[22] Silveira EA , da Silva Filho RR , Spexoto MCB , Haghighatdoost F , Sarrafzadegan N , de Oliveira C . The role of sarcopenic obesity in cancer and cardiovascular disease: a synthesis of the evidence on pathophysiological aspects and clinical implications. Int J Mol Sci 2021;22:22. 10.3390/ijms22094339 PMC8122649 33919368

[23] Sahakyan KR , Somers VK , Rodriguez‐Escudero JP , Hodge DO , Carter RE , Sochor O , et al. Normal‐weight central obesity: implications for total and cardiovascular mortality. Ann Intern Med 2015;163:827–835. 26551006 10.7326/M14-2525 PMC4995595

[24] Janssen I , Heymsfield SB , Allison DB , Kotler DP , Ross R . Body mass index and waist circumference independently contribute to the prediction of nonabdominal, abdominal subcutaneous, and visceral fat. Am J Clin Nutr 2002;75:683–688. 11916754 10.1093/ajcn/75.4.683

[25] Ohlson LO , Larsson B , Svärdsudd K , Welin L , Eriksson H , Wilhelmsen L , et al. The influence of body fat distribution on the incidence of diabetes mellitus. 13.5 years of follow‐up of the participants in the study of men born in 1913. Diabetes 1985;34:1055–1058. 4043554 10.2337/diab.34.10.1055

[26] Rexrode KM , Carey VJ , Hennekens CH , Walters EE , Colditz GA , Stampfer MJ , et al. Abdominal adiposity and coronary heart disease in women. JAMA 1998;280:1843–1848. 9846779 10.1001/jama.280.21.1843

[27] Després JP . Intra‐abdominal obesity: an untreated risk factor for Type 2 diabetes and cardiovascular disease. J Endocrinol Invest 2006;29:77–82. 16751711

[28] Shirasawa T , Ochiai H , Yoshimoto T , Nagahama S , Kobayashi M , Ohtsu I , et al. Associations between normal weight central obesity and cardiovascular disease risk factors in Japanese middle‐aged adults: a cross‐sectional study. J Health Popul Nutr 2019;38:46. 31849344 10.1186/s41043-019-0201-5 PMC6918653

[29] Manolopoulos KN , Karpe F , Frayn KN . Gluteofemoral body fat as a determinant of metabolic health. Int J Obes (Lond) 2010;34:949–959. 20065965 10.1038/ijo.2009.286

[30] Kaneko H , Yano Y , Lee H , Lee HH , Okada A , Suzuki Y , et al. Blood pressure classification using the 2017 ACC/AHA guideline and heart failure in patients with cancer. J Clin Oncol 2023;41: 980–990, 990. 36075006 10.1200/JCO.22.00083

[31] Yamana H , Moriwaki M , Horiguchi H , Kodan M , Fushimi K , Yasunaga H . Validity of diagnoses, procedures, and laboratory data in Japanese administrative data. J Epidemiol 2017;27:476–482. 28142051 10.1016/j.je.2016.09.009 PMC5602797

[32] Fujihara K , Yamada‐Harada M , Matsubayashi Y , Kitazawa M , Yamamoto M , Yaguchi Y , et al. Accuracy of Japanese claims data in identifying diabetes‐related complications. Pharmacoepidemiol Drug Saf 2021;30:594–601. 33629363 10.1002/pds.5213

[33] Saito I , Yamagishi K , Kokubo Y , Yatsuya H , Iso H , Sawada N , et al. Association between mortality and incidence rates of coronary heart disease and stroke: the Japan Public Health Center‐based prospective (JPHC) study. Int J Cardiol 2016;222:281–286. 27497111 10.1016/j.ijcard.2016.07.222

[34] Miura K , Nakagawa H , Ohashi Y , Harada A , Taguri M , Kushiro T , et al. Four blood pressure indexes and the risk of stroke and myocardial infarction in Japanese men and women: a meta‐analysis of 16 cohort studies. Circulation 2009;119:1892–1898. 19332464 10.1161/CIRCULATIONAHA.108.823112

