# Supplementary material for: Metabolic syndrome and cardiovascular disease in cancer survivors
Source: J Cachexia Sarcopenia Muscle. 2024 Mar 22;15(3):1062–71. doi: 10.1002/jcsm.13443 (PMC11154793; doi:10.1002/jcsm.13443)
Supplement: Supplementary file 1 — Table S1. The International Diabetes Federation's criteria and the National Cholesterol Education Program Adult Treatment Panel III criteria for defining metabolic syndrome. Table S2. Metabolic Syndrome and Each Cardiovascular Event. Table S3. The Frequency of Events, Corresponding Incidence Rates, and Hazard Ratios for Cardiovascular Disease Events Among Participants by Metabolic Syndrome Defined using the International Diabetes Federation criteria. Table S4. The Frequency of Events, Corresponding Incidence Rates, and Hazard Ratios for Cardiovascular Disease Events Among Participants by Metabolic Syndrome Defined using the National Cholesterol Education Program Adult Treatment Panel III criteria. Table S5. Multiple Imputation. Table S6. Analysis after the exclusion of subjects with follow‐up period shorter than one year. Table S7. Competing Risks Analysis. [file JCSM-15-1062-s002.docx]

| **Supplementary Table 1. The International Diabetes Federation’s criteria and the National Cholesterol Education Program Adult Treatment Panel III criteria for defining metabolic syndrome** | |
| --- | --- |
| **The International Diabetes Federation’s criteria for defining metabolic syndrome** | |
| High waist circumference plus any two or three of the following: | Categorical cut points |
| High waist circumference | ≥ 90 cm for men  or ≥ 80 cm for women |
| High blood pressure | Systolic blood pressure ≥ 130 mmHg or diastolic blood pressure ≥ 85 mmHg or use of antihypertensive medications |
| Hyperglycemia | Fasting plasma glucose level ≥ 100 mg/dL or use of antidiabetic medications |
| Dyslipidemia | Triglycerides level ≥ 150 mg/dL or high-density lipoprotein cholesterol level < 40 mg/dL for men and < 50 mg/dL for women or use of lipid-lowering medications |
| **The National Cholesterol Education Program Adult Treatment Panel III criteria for defining metabolic syndrome** | |
| Any three, four, or five of the following: | Categorical cut points |
| High waist circumference | ≥ 102 cm for men or ≥ 88 cm for women |
| High blood pressure | Systolic blood pressure ≥ 130 mmHg or diastolic blood pressure ≥ 85 mmHg or use of antihypertensive medications |
| Hyperglycemia | Fasting plasma glucose level ≥ 100 mg/dL or use of antidiabetic medications |
| Elevated triglycerides | Triglycerides level ≥ 150 mg/dL or use of lipid-lowering medications |
| Reduced high-density lipoprotein cholesterol | High-density lipoprotein cholesterol level < 40 mg/dL for men and < 50 mg/dL for women or use of lipid-lowering medications |
|  | |

| **Supplementary Table 2. Metabolic Syndrome and Each Cardiovascular Event** | | | | | | | |
| --- | --- | --- | --- | --- | --- | --- | --- |
| Outcome | Metabolic Syndrome | No | No. of Events | Incidence Rate (95% CI) | Model 1 | Model 2 | Model 3 |
| Heart Failure | Absent | 47,952 | 1,292 | 97.8 (92.6-103.3) | 1 [Reference] | 1 [Reference] | 1 [Reference] |
|  | Present | 5,558 | 255 | 185.9 (164.4-210.1) | 1.91 (1.67- 2.18) | 1.51 (1.32- 1.74) | 1.24 (1.05- 1.45) |
| Myocardial Infarction | Absent | 47,952 | 92 | 6.8 (5.6-8.4) | 1 [Reference] | 1 [Reference] | 1 [Reference] |
|  | Present | 5,558 | 28 | 19.8 (13.7-28.7) | 2.94 (1.93- 4.50) | 2.03 (1.30- 3.16) | 2.01 (1.20- 3.39) |
| Angina Pectoris | Absent | 47,952 | 1,096 | 83.0 (78.2-88.0) | 1 [Reference] | 1 [Reference] | 1 [Reference] |
|  | Present | 5,558 | 211 | 153.6 (134.2-175.7) | 1.84 (1.59- 2.14) | 1.49 (1.28- 1.74) | 1.36 (1.14- 1.62) |
| Stroke | Absent | 47,952 | 584 | 43.8 (40.4-47.5) | 1 [Reference] | 1 [Reference] | 1 [Reference] |
|  | Present | 5,558 | 118 | 84.5 (70.6-101.3) | 1.93 (1.58- 2.35) | 1.45 (1.18- 1.78) | 1.43 (1.13- 1.82) |
| The incidence rate was per 10,000 person-years. Unadjusted and adjusted hazard ratios (95% CI) associated with metabolic syndrome are shown. Model 1 is unadjusted. Model 2 includes adjustments for age and sex. Model 3 includes adjustment for age, sex, body mass index, low-density lipoprotein cholesterol level, current cigarette smoking, alcohol drinking, physical inactivity, cancer sites, and active cancer treatment before and after 6 months. Patients were categorized into two groups according to the absence or presence of the metabolic syndrome. CI=confidence interval. | | | | | | | |

| **Supplementary Table 3. The Frequency of Events, Corresponding Incidence Rates, and Hazard Ratios for Cardiovascular Disease Events Among Participants by Metabolic Syndrome Defined using the International Diabetes Federation criteria** | | | | | | |
| --- | --- | --- | --- | --- | --- | --- |
|  | Overall | | Men | | Women | |
| Variable | Metabolic Syndrome | | Metabolic Syndrome | | Metabolic Syndrome | |
|  | Absent | Present | Absent | Present | Absent | Present |
| Number | 45,731 | 7,779 | 16,845 | 3,248 | 28,886 | 4,531 |
| CVD Events | 2,456 | 629 | 1,157 | 301 | 1,299 | 328 |
| Incidence Rate (95% CI) | 197.6 (190.0-205.6) | 343.9 (318.0-371.8) | 246.7 (232.8-261.3) | 387.5 (346.1-433.8) | 167.9 (159.0-177.3) | 311.7 (279.7-347.3) |
| Model 1 (Unadjusted) | 1 [Reference] | 1.73 (1.59- 1.89) | 1 [Reference] | 1.57 (1.38- 1.78) | 1 [Reference] | 1.85 (1.64- 2.09) |
| Model 2 | 1 [Reference] | 1.48 (1.36- 1.62) | 1 [Reference] | 1.52 (1.34- 1.73) | 1 [Reference] | 1.46 (1.29- 1.65) |
| Model 3 | 1 [Reference] | 1.34 (1.21- 1.49) | 1 [Reference] | 1.47 (1.26- 1.73) | 1 [Reference] | 1.27 (1.10- 1.46) |
| The incidence rate was per 10,000 person-years. Unadjusted and adjusted hazard ratios (95% CI) associated with metabolic syndrome are shown. Model 1 is unadjusted. Model 2 includes adjustments for age and sex. Model 3 includes adjustment for age, sex, body mass index, low-density lipoprotein cholesterol level, current cigarette smoking, alcohol drinking, physical inactivity, cancer sites, and active cancer treatment before and after 6 months. In the analysis stratified by sex, sex was excluded from covariates. Patients were categorized into two groups according to the absence or presence of the metabolic syndrome. CVD=cardiovascular disease; CI=confidence interval. | | | | | | |

| **Supplementary Table 4. The Frequency of Events, Corresponding Incidence Rates, and Hazard Ratios for Cardiovascular Disease Events Among Participants by Metabolic Syndrome Defined using the National Cholesterol Education Program Adult Treatment Panel III criteria** | | | | | | |
| --- | --- | --- | --- | --- | --- | --- |
|  | Overall | | Men | | Women | |
| Variable | Metabolic Syndrome | | Metabolic Syndrome | | Metabolic Syndrome | |
|  | Absent | Present | Absent | Present | Absent | Present |
| Number | 46,225 | 7,285 | 16,461 | 3,632 | 29,764 | 3,653 |
| CVD Events | 2,476 | 609 | 1,130 | 328 | 1,346 | 281 |
| Incidence Rate (95% CI) | 197.4 (189.7-205.3) | 356.2 (329.0-385.6) | 246.1 (232.2-260.9) | 374.5 (336.1-417.3) | 169.2 (160.4-178.5) | 336.9 (299.7-378.7) |
| Model 1 (Unadjusted) | 1 [Reference] | 1.80 (1.65- 1.97) | 1 [Reference] | 1.52 (1.34- 1.72) | 1 [Reference] | 1.98 (1.74- 2.26) |
| Model 2 | 1 [Reference] | 1.46 (1.33- 1.60) | 1 [Reference] | 1.39 (1.22- 1.57) | 1 [Reference] | 1.56 (1.36- 1.78) |
| Model 3 | 1 [Reference] | 1.32 (1.19- 1.46) | 1 [Reference] | 1.29 (1.13- 1.48) | 1 [Reference] | 1.36 (1.16- 1.58) |
| The incidence rate was per 10,000 person-years. Unadjusted and adjusted hazard ratios (95% CI) associated with metabolic syndrome are shown. Model 1 is unadjusted. Model 2 includes adjustments for age and sex. Model 3 includes adjustment for age, sex, body mass index, low-density lipoprotein cholesterol level, current cigarette smoking, alcohol drinking, physical inactivity, cancer sites, and active cancer treatment before and after 6 months. In the analysis stratified by sex, sex was excluded from covariates. Patients were categorized into two groups according to the absence or presence of the metabolic syndrome. CVD=cardiovascular disease; CI=confidence interval. | | | | | | |

| **Supplementary Table 5. Multiple Imputation** | | | | | | | |
| --- | --- | --- | --- | --- | --- | --- | --- |
| Outcome | Metabolic Syndrome | No | No. of Events | Incidence Rate (95% CI) | Model 1 | Model 2 | Model 3 |
| Cardiovascular disease | Absent | 55,442 | 3,117 | 204.3 (197.2-211.5) | 1 [Reference] | 1 [Reference] | 1 [Reference] |
|  | Present | 6,508 | 607 | 380.3 (351.2-411.8) | 1.86 (1.70-2.02) | 1.49 (1.36-1.63) | 1.31 (1.18-1.46) |
| The incidence rate was per 10,000 person-years. Unadjusted and adjusted hazard ratios (95% CI) associated with metabolic syndrome are shown. Model 1 is unadjusted. Model 2 includes adjustments for age and sex. Model 3 includes adjustment for age, sex, body mass index, low-density lipoprotein cholesterol level, current cigarette smoking, alcohol drinking, physical inactivity, cancer sites, and active cancer treatment before and after 6 months. Patients were categorized into two groups according to the absence or presence of the metabolic syndrome. CI=confidence interval. | | | | | | | |

| **Supplementary Table 6. Analysis after the exclusion of subjects with follow-up period shorter than one year** | | | | | | | |
| --- | --- | --- | --- | --- | --- | --- | --- |
| Outcome | Metabolic Syndrome | No | No. of Events | Incidence Rate (95% CI) | Model 1 | Model 2 | Model 3 |
| Cardiovascular disease | Absent | 36,450 | 1,704 | 194.3 (185.3-203.7) | 1 [Reference] | 1 [Reference] | 1 [Reference] |
|  | Present | 4,000 | 317 | 369.3 (330.8-412.3) | 1.90 (1.69- 2.14) | 1.50 (1.32- 1.70) | 1.33 (1.15- 1.53) |
| The incidence rate was per 10,000 person-years. Unadjusted and adjusted hazard ratios (95% CI) associated with metabolic syndrome are shown. Model 1 is unadjusted. Model 2 includes adjustments for age and sex. Model 3 includes adjustment for age, sex, body mass index, low-density lipoprotein cholesterol level, current cigarette smoking, alcohol drinking, physical inactivity, cancer sites, and active cancer treatment before and after 6 months. Patients were categorized into two groups according to the absence or presence of the metabolic syndrome. CI=confidence interval | | | | | | | |

| **Supplementary Table 7. Competing Risks Analysis** | | | | | | | |
| --- | --- | --- | --- | --- | --- | --- | --- |
| Outcome | Metabolic Syndrome | No | No. of Events | Incidence Rate (95% CI) | Model 1 | Model 2 | Model 3 |
| Cardiovascular disease | Absent | 47,952 | 2,596 | 200.8 (193.2-208.7) | 1 [Reference] | 1 [Reference] | 1 [Reference] |
|  | Present | 5,558 | 489 | 368.2 (336.9-402.3) | 1.82 (1.65-2.01) | 1.47 (1.32-1.62) | 1.29 (1.15-1.45) |
| The incidence rate was per 10,000 person-years. Unadjusted and adjusted hazard ratios (95% CI) associated with metabolic syndrome are shown. Model 1 is unadjusted. Model 2 includes adjustments for age and sex. Model 3 includes adjustment for age, sex, body mass index, low-density lipoprotein cholesterol level, current cigarette smoking, alcohol drinking, physical inactivity, cancer sites, and active cancer treatment before and after 6 months. Patients were categorized into two groups according to the absence or presence of the metabolic syndrome. CI=confidence interval | | | | | | | |
