# Supplementary material for: Metabolic syndrome and cardiovascular disease in cancer survivors
Source: J Cachexia Sarcopenia Muscle. 2024 Mar 22;15(3):1062–71. doi: 10.1002/jcsm.13443 (PMC11154793; doi:10.1002/jcsm.13443)
Supplement: Supplementary file 2 — Figure S1. Study Design. Figure S2. Flowchart. [file JCSM-15-1062-s001.pptx]

## Slide 1
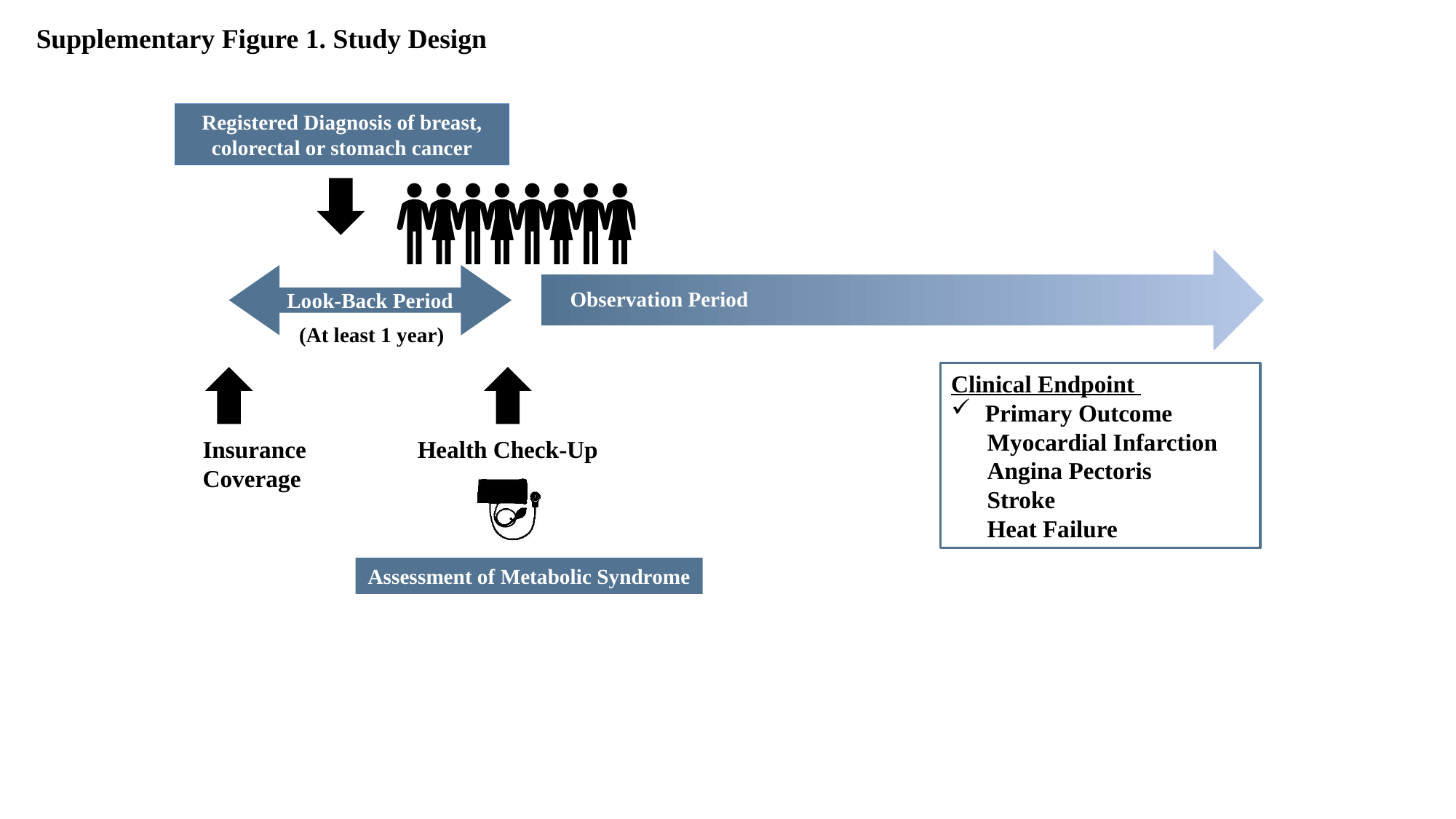

Supplementary Figure 1. Study Design
Registered Diagnosis of breast, colorectal or stomach cancer
Observation Period
Look-Back Period
(At least 1 year)
Clinical Endpoint
Primary Outcome
 Myocardial Infarction
 Angina Pectoris
 Stroke
 Heat Failure
Health Check-Up
Insurance
Coverage
Assessment of Metabolic Syndrome

## Slide 2
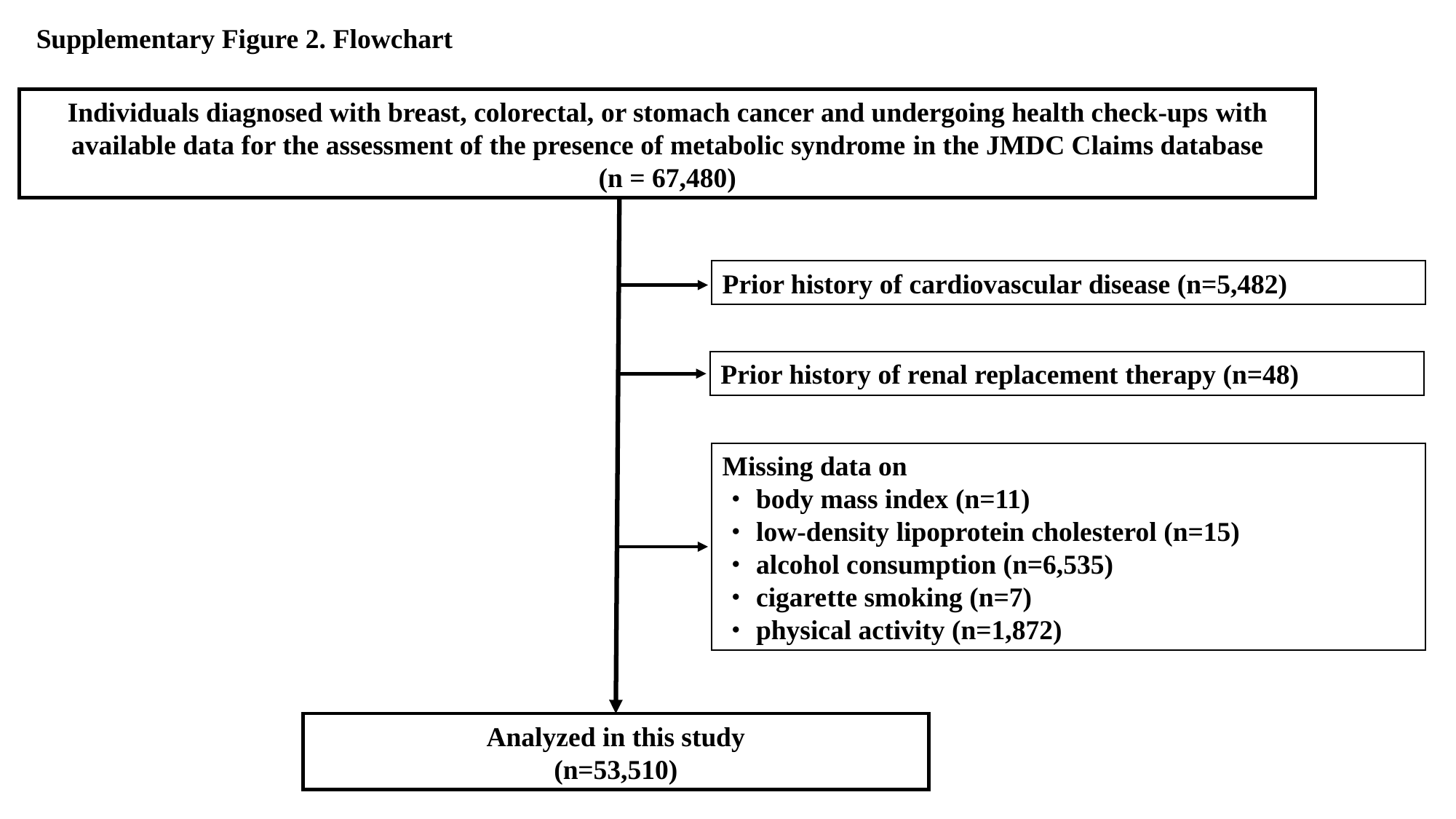

Supplementary Figure 2. Flowchart
Individuals diagnosed with breast, colorectal, or stomach cancer and undergoing health check-ups with available data for the assessment of the presence of metabolic syndrome in the JMDC Claims database
(n = 67,480)
Prior history of cardiovascular disease (n=5,482)
Prior history of renal replacement therapy (n=48)
Missing data on
・body mass index (n=11)
・low-density lipoprotein cholesterol (n=15)
・alcohol consumption (n=6,535)
・cigarette smoking (n=7)
・physical activity (n=1,872)
Analyzed in this study
(n=53,510)
